# The Impact of Pulsed Electric Field on the Extraction of Bioactive Compounds from Beetroot

**DOI:** 10.3390/foods8070244

**Published:** 2019-07-05

**Authors:** Malgorzata Nowacka, Silvia Tappi, Artur Wiktor, Katarzyna Rybak, Agnieszka Miszczykowska, Jakub Czyzewski, Kinga Drozdzal, Dorota Witrowa-Rajchert, Urszula Tylewicz

**Affiliations:** 1 Department of Food Engineering and Process Management, Faculty of Food Sciences, Warsaw University of Life Sciences, Nowoursynowska 159c, 02-776 Warsaw, Poland; 2 Interdepartmental Centre for Agri-Food Industrial Research, University of Bologna, Via Quinto Bucci 336, 47521 Cesena, Italy; 3 Department of Agricultural and Food Sciences, University of Bologna, Piazza Goidanich 60, 47521 Cesena, Italy

**Keywords:** pulsed electric field, extraction, bioactive compounds, red beet

## Abstract

Beetroot is a root vegetable rich in different bioactive components, such as vitamins, minerals, phenolics, carotenoids, nitrate, ascorbic acids, and betalains, that can have a positive effect on human health. The aim of this work was to study the influence of the pulsed electric field (PEF) at different electric field strengths (4.38 and 6.25 kV/cm), pulse number 10–30, and energy input 0–12.5 kJ/kg as a pretreatment method on the extraction of betalains from beetroot. The obtained results showed that the application of PEF pre-treatment significantly (*p* < 0.05) influenced the efficiency of extraction of bioactive compounds from beetroot. The highest increase in the content of betalain compounds in the red beet’s extract (betanin by 329%, vulgaxanthin by 244%, compared to the control sample), was noted for 20 pulses of electric field at 4.38 kV/cm of strength. Treatment of the plant material with a PEF also resulted in an increase in the electrical conductivity compared to the non-treated sample due to the increase in cell membrane permeability, which was associated with leakage of substances able to conduct electricity, including mineral salts, into the intercellular space.

## 1. Introduction 

Beetroot (*Beta vulgaris* L.) is part of the Chenopodiaceae family and has originated in Asia and Europe. The red beetroot variety, a cultivated form of *Beta vulgaris* subsp. *vulgaris* (conditiva), is widely used all over the world to produce pickles, salad, or juice [1]. Beetroot contains many functional components, such as vitamins, minerals (potassium, sodium, phosphorous, calcium, magnesium, copper, iron, zinc, manganese), phenolics, carotenoids, nitrate, and ascorbic acids that promote health benefits [2]. In fact, polyphenols, carotenoids, and vitamins present in beetroot have been recognized to have antioxidant, anti-inflammatory, anticarcinogenic, and hepato-protective activities, which can help in the prevention of many diseases, such as cardiovascular disease or hypertension and diabetes [3]. 

The characteristic red purple color of beetroot derives from betalain, water soluble pigments found in plants of the Caryophyllales order [4]. Depending on their chemical structure, betalains can be divided into red-purple and violet betacyanins (betanin, isobetanin, probetanin, and neobetanin) or yellow betaxanthins (vulgaxanthin, miraxanthin, portulaxanthin, and indicaxanthin) [5]; and the redness of beetroot depends on the ratio between the two classes [6]. For example, it was observed that the intact beetroot plant extracts contain about 40 mg/g dm of betalains, from which 20.75 mg/g dm are betacyanins and 19.01 mg/g dm are betaxanthins [2]. Slavov et al. [3] studied the individual betalain compounds content in pressed juice from beetroot, showing that the most abundant were betanin, followed by vulgaxanthin and isobetanin.

Betalain pigments can generally exhibit health benefits as antioxidants, anti-cancer, anti-lipidemic, and antimicrobial agents [5,7]. They are mostly used as food dyes due to non-precarious, non-toxic, non-carcinogenic, and non-poisonous nature [2]. Therefore, there is an increasing interest of food industries in the extraction of this natural food colorant. In order to extract the pigments from plant material, the disruption of membranes is necessary, and this is usually obtained through the application of detergents, solvents, or thermal treatments [8]. The latter is the preferred method for color concentrates production; however, betalain is highly sensitive to heat. Therefore, alternative methods are necessary in order to prevent the discoloration of the pigments [9]. In recent years, the use of novel and mild methods has been investigated, including, among others, the microwave [9,10] and ultrasound [11,12] assisted extraction . Another promising technique could be application of pulsed electric field (PEF) that, applied as a pre-treatment for the extraction, allows the selective recovery of bioactive compounds, at the same time reducing the energy and time required in the process. 

PEF is the application of short time pulsed with high voltage into the food product placed between two electrodes [13,14], thus promoting the modification of membrane permeability and the increase of the extraction yield [15,16].

Recently, PEF treatment has been applied in order to recover pigments from beetroot. Fincan et al. [17] observed a release of about 90% of total red coloring from beetroot subjected to 270 rectangular pulses of 10 μs at 1 kV/cm field strength and total energy of 7 kJ/kg. López et al. [18] and Luengo et al. [19] studied the influence of different PEF parameters, such as electric field strength, pulse number, pulse duration, and specific energy applied on the extractability of betanin from the beetroot cylinders. Similarly, Chalermchat et al. [20] evaluated the impact of PEF treatment on the red pigment in beetroot discs. However, in the cited works, PEF was applied only on a single geometry (cylinder or disc), while we assumed that this parameter could also have an influence on the extraction. Moreover, in most cases, the efficiency of extraction was assessed only by measuring the difference in the color of the extracts. Hence, our goals in the present research were to compare (i) the impact of mechanical preparation (cylinders or pulp) and (ii) the impact of PEF pre-treatments on different geometric forms on extractability of beetroot pigments. For these aims, beetroot was subjected to cutting into cylinders or pulping and different PEF conditions (electric field strength between 4.38 and 6.25 kV/cm, pulse number in the range of 10–30, energy input in the range of 0–12.5 kJ/kg) as pre-treatments for the extraction of selected red (betanin) and yellow (vulgaxanthin) pigments. 

## 2. Materials and Methods

### 2.1. Preparation of Samples

Beetroot (*Beta vulgaris* L.) was purchased from a local market (Warsaw, Poland). Products with a similar degree of maturity and characterized by similar dimensions were selected for the study. The raw materials were stored at 4 °C until the start of the experiments (not longer than one week). Before each experiment, the vegetables were removed from the cooling chamber, allowed to reach room temperature, and then washed. The beetroot was cut into 7 mm height slices, and then cylinders with a diameter of d = 15 mm were obtained using a cork borer. An electric mill was used to crush the cylinders to obtain a pulp.

### 2.2. PEF Treatement

A prototype PEF generator ERTEC-RI-1B (ERTEC, Wroclaw, Poland) with output high-voltage impulse up to 30 kV and capacitance of 0.25 μF reactor was used. The generator provided monopolar, exponential shaped pulses with an average width of 10 μs and an interval between pulses equal to 2 s, which allows minimization of the temperature increase during the electric field application. The treatment chamber (diameter of 40 mm, height of 16 mm), made of dielectric material (Corian) and two stainless-steel lids, consists of a spark gap controlling the flow of electrical impulses and a set of electrodes (stationary and mobile). Both stationary and mobile electrodes were connected to the PEF generator.

Beetroot samples (8 cylinders) were placed in the treatment chamber. Next, to ensure a good contact between the electrodes and the tested material, the chamber was filled with a phosphate buffer at pH = 6.5 and closed using a mobile electrode. In the case of pulp, the entire volume of the electrical processing chamber (20 cm^3^) was carefully filled with the sample without additional solvent. PEF application was carried out using two different electric field intensities (4.38 and 6.25 kV/cm) and three pulse numbers (10, 20, 30). Each combination of the experiment was performed in three repetitions. The specific energy intake was calculated according to the following equation [14]:

*W_s_* = (*V^2^Cn*)/(*2m*), (1)

where *V* (V) is the voltage, *C* (F) is a capacitance of the energy storage capacitor, *n* is number of pulses, and *m* (kg) is mass of the sample in the treatment chamber. 

The energy values (*W_s_*) supplied to the sample are shown in Table 1. After PEF treatment, cylinder samples were dried on filter paper. Directly after PEF application, the temperature increase of the samples did not exceed 9.2 °C. 

### 2.3. Electrical Conductivity

The electrical conductivity of the sample was measured using a conductometer (CPC-505, Elmetron, Gliwice, Poland) with a platinum dual-needle probe, and it was used to evaluate the effectiveness of electroporation. The electrical conductivity was measured continuously for at least 5 min at room temperature. The measurements were conducted in five repetitions [21].

### 2.4. Color Determination

The color of the beetroot cylinders and extracts were measured in the CIE L*a*b* color system using a Konica Minolta CR-5 Chroma Meter (Osaka, Japan) with D65 light source and standard 2º observer. The L* parameter defines the lightness of the sample, the a* chromatic coordinate determines the green (−) and red (+) component, and the b* coordinate describes the blue (−) and yellow (+) component [22]. The measurements were conducted at sixteen repetitions. Moreover, the supernatant (solution remaining after extraction) was subjected to the color measurements in the transmittance mode. 

### 2.5. Betalain Content

The betalain content in raw and PEF-treated material was determined by the chemical method, which is based on the extraction of dyes using phosphate buffer (pH 6.5) and the simultaneous determination of red and yellow dyes measured spectrophotometrically [23]. In the case of raw (untreated) material and beetroot pulp, 0.5 g of sample was weighted and then quantitatively transferred to a falcon tube, added a 50 cc phosphate buffer, and placed on a Vortex shaker for 20 min at 2000 rpm. After shaking, the obtained supernatant was filtered and the absorbance of the solution was measured in a spectrophotometer Heλios ThermoSpectronic γ (Thermo Electron Corporation, USA) at 476, 538, and 600 nm using a phosphate buffer solution as standard. In the case of PEF-treated material, a single beetroot cylinder along with the phosphate buffer was quantitatively transferred to the falcon. Each time, the PEF chamber was rinsed three times with buffer and added to the falcon in order to avoid betalain losses. The falcon was filled with a buffer to the 50 cc volume and then analyzed as described above. The assay was performed three times. 

The absorbance value for red dyes (B)] at λ = 538 nm including light absorption due to the presence of impurities was calculated as:

*E_B_ = 1.095 × (E_538_ − E_600_)*, (2)

where *E_538_* absorbance at λ = 538 nm, *E_600_* absorbance at λ = 600 nm, and *1.095* is a absorption coefficient at λ = 538 nm resulting from impurities present. 

The content of red dyes (B) (mg betanin/100 g dm) was calculated as:

*B = (c × E_B_)/(1120 × a × b)*, (3)

where *c* (mg) mass of the sample with buffer, *1120* (-) value resulting from the absorbance of a 1% betanin solution measured at 538 nm in a 1 cm cuvette, *a* (g) sample weight, and *b* (g/g dm) is dry matter content.

The absorbance value for yellow dyes (W) at λ = 476 nm including light absorption due to the presence of impurities and red dyes and was calculated as:

*E_W_ = E_476_ − E_358_ + 0.667 × E_B_*, (4)

where *E_476_* absorbance at λ = 476 nm, *E_538_* absorbance at λ = 538 nm, and *E_B_* is the absorbance value for red pigments.

The content of yellow dyes (W) (mg vulgaxanthin /100 g dm) was calculated as:

*W = (c × E_W_)/(750 × a × b)*, (5)

where *c* (mg) mass of the sample with buffer, *750* (-) value resulting from the absorbance of a 1% betanin solution measured at 476 nm in a 1 cm cuvette, *a* (g) sample weight, and *b* (g/g dm) is dry matter content. 

The content of red and yellow dyes ware quantified as mg of betanin and vulgaxanthin, respectively, in 100 g dry material.

### 2.6. Statitical Analysis—PCA and Pearson’s Correlation

The ANOVA and the Tukey test (α = 0.05) were applied to evaluate significant differences between investigated samples. The two-way ANOVA and comparison of obtained partial η^2^ values have been used to assess the effect size of the analyzed variables on the electrical conductivity. The Pearson’s correlation coefficient was calculated in order to evaluate the dependence between selected parameters of analyzed tissue. Hierarchical Cluster Analysis (HCA) with Ward’s agglomeration method was done for complex assessment of obtained data. Euclidian distance was used to express obtained results by the means of relative distance. All calculations were performed using STATISTICA 13 (StatSoft Inc., Washington, DC, USA) and Excel (Microsoft, New York, NY, USA) software. 

## 3. Results and Discussion

### 3.1. Electical Conductivity 

**Figure 1:**
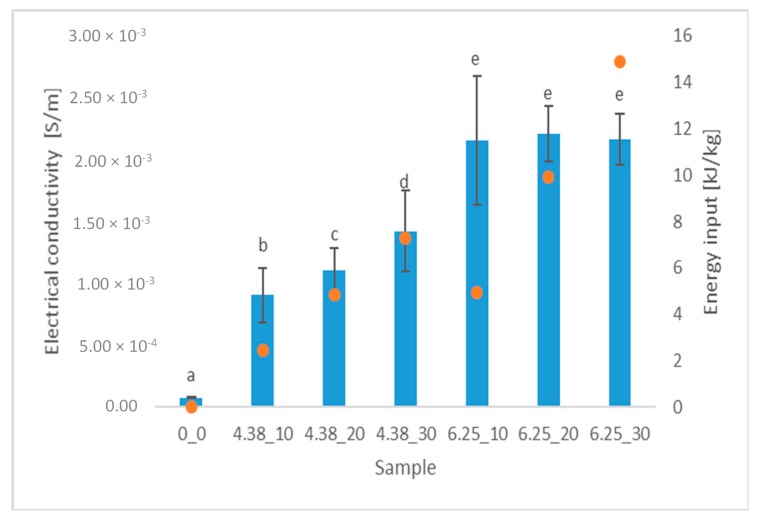
Electrical conductivity S/m (bars) and total energy input kJ/kg (dots) of differently treated beetroot samples. The same letter on the same column means no significant difference between electrical conductivities by the Tukey test (*p* < 0.05).

Electrical conductivity measurement is used to evaluate the effectiveness of PEF treatment in biological tissue [24]. Following electroporation and intracellular content leakage, electrical conductivity of plant materials rises, indicating cell membrane rupture [25]. Figure 1 shows the results of both electrical conductivity and energy input delivered to the beetroot samples. As expected, all PEF pre-treated samples exhibited higher electrical conductivity in comparison to intact tissue. For instance, material treated by 10 pulses at 4.38 kV/cm was characterized by electrical conductivity equal to 9.05 × 10^−4^ S/m. When the number of pulses increased to 30 (hence increasing the energy input) at the same electric field intensity, the electrical conductivity rose significantly (*p* < 0.05) to 14.2 × 10^−4^ S/m. The application of the higher electric field intensity (6.25 kV/cm) lead to a further increase of electrical conductivity. However, in this case, the difference between samples pre-treated by 10, 20, and 30 pulses were statistically insignificant (*p* > 0.05). In other words, despite the energy input increase, the electroporation efficiency did not grow because of the saturation of the electroporation phenomenon that was previously observed in the literature for materials other than beetroot [14,26,27]. 

The performed statistical analysis showed that electric field intensity and number of pulses significantly (*p* < 0.05) influenced the electrical conductivity of the beetroot samples. Moreover, the interaction of these parameters, which could be interpreted as the influence of energy input, also had a significant (*p* < 0.05) impact on the electroporation efficiency measured by electrical conductivity measurement. However, comparing the partial η^2^ values, it can be stated that the biggest size effect was found for electric field intensity (η^2^ = 0.753). For comparison, partial η^2^ was equal to 0.0114 and 0.113, for number of pulses and the interaction between electric field intensity and number of pulses, respectively. Similar results were obtained previously by Wiktor et al. [21], suggesting that the cell disintegration index could be influenced not only by electric field strength and total energy input but also by the pulses number applied during the treatment, simply because it increases the chance of electroporation to occur.

The PEF parameters that have been used in further analysis were selected in order to allow to study the extraction of pigments from (i) samples that have been treated with similar energy input but manifested different electrical conductivity (4.38_20 and 6.25_10) and (ii) samples that exhibited similar electroporation efficiency (expressed as similar electrical conductivity) but treated with different energy input (6.25_10 and 6.25_30).

### 3.2. Color Determination

Color is a major quality index for fresh and processed fruit and vegetables, being one of the main influencing parameters for consumers. Moreover, color could be also indicator of the presence and the quantity of bioactive compounds, e.g., betanins which are responsible for the characteristic red-purple color of the beetroot [4].

Table 2 reports the color parameters of untreated and PEF-treated beetroot tissue before and after extraction. It can be observed that the application of PEF decreased significantly all the color parameters, and this decrease was more accentuated in the samples 4.38_20 and 6.25_30. The decrease of a* and b* values indicates the loss of the pigments (red betanin and yellow vulgaxanthin) from the beetroot to the extracting solution. 

The application of 4.38 kV/cm caused a significant decrease in both parameters in the beetroot tissue after extraction, while for other PEF conditions these values were mostly unchanged. When comparing the color parameters before and after extraction, it is visible that cylinders after extraction exhibited higher values of L*, a* and b*. Such a situation can be linked to the diffusion of pigments from the inner parts of the tissue towards its surface during extraction and by lower water content after extraction which influenced the light reflection during measurement.

**Figure A1:**
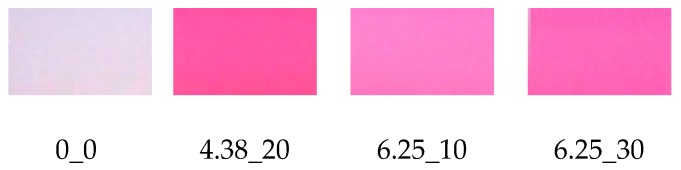
Changes in the color of the extract from the beetroot slices depending on the parameters of the applied pulsed electric field treatment.

Table 3 shows the color parameters of beetroot extracts obtained from untreated and PEF-treated beetroot cylinders and pulp. It is possible to observe a significant (*p* < 0.05) decrease in the lightness and a significant increase of the red color component of the beetroot cylinders subjected to all PEF treatments, without significant differences due to treatment parameters (Figure A1). The color intensity is usually directly related to the content of betalains extracted from beets. The use of PEF clearly increases the color intensity, and therefore it increases the concentration of these compounds [28]. Concerning the yellow index, a decrease was observed only in samples treated at 6.25 kV/cm, probably due to the degradation of the yellow dye—vulgaxanthin. 

Concerning the extract obtained from the pulped beetroot (Table 3), it was possible to observe that the pulping process itself caused a significant reduction in the brightness of the extract, as well as an increase in the share of red color. The application of PEF did not result in significant differences between the values of the L* and a* parameters. On the other side, the b* coordinate was characterized by a lower value in samples treated at 6.25 kV/cm, further reduced increasing pulses from 10 to 30, probably indicating a lower amount of vulgaxanthin in the extract.

### 3.3. Betalain Content

**Figure 2:**
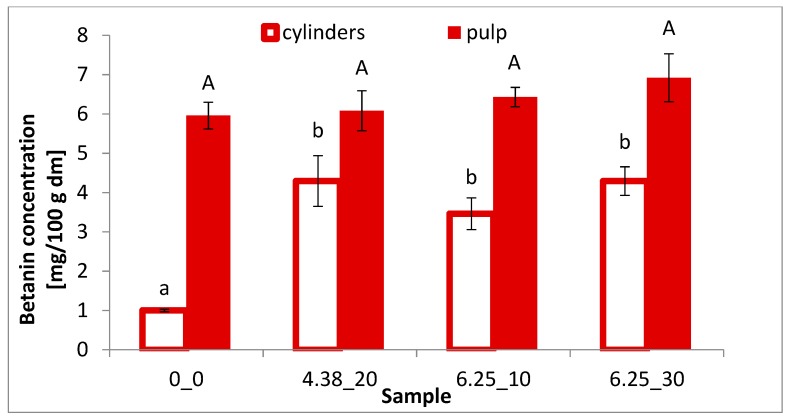
Betanin concentration of differently treated beetroot cylinders and pulp. The same letter (and the same letter size) on the same column means no significant difference by the Tukey test (*p* < 0.05).

**Figure 3:**
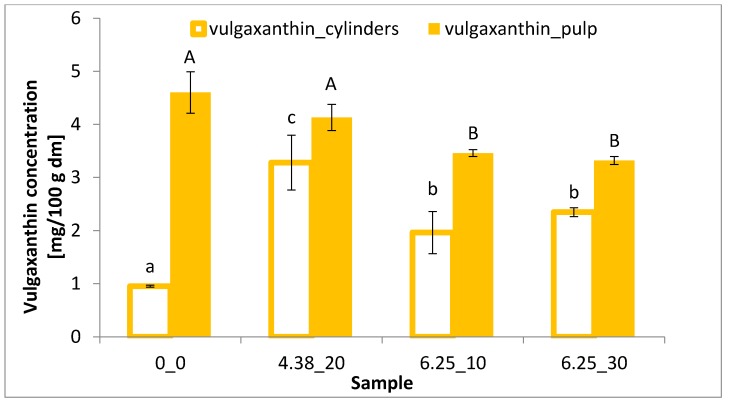
Vulgaxanthin concentration of differently treated beetroot cylinders and pulp. The same letter (and the same letter size) on the same column means no significant difference by the Tukey test (*p* < 0.05).

Betanin and vulgaxanthin are the most abundant betalains in beetroot [3]. Figure 2 shows the betanin content, while Figure 3 shows the vulgaxanthin content of differently treated beetroot samples. The application of PEF to beetroot cylinders allowed to increase the extraction of betanin in a similar way (of about 3-fold) for all the PEF conditions applied. The use of PEF at the intensity of 4.38 kV/cm significantly (*p* < 0.05) increased the yield of betanin and vulgaxanthin extraction from beetroot cylinders by 329% and 244%, respectively, compared to the control samples. This increase indicates that PEF facilitates their extraction by increasing cell membrane permeability, which is also confirmed by the color parameters. While the values of the red and yellow color components decreased in the cylinders after extraction, an increase was recorded in the post-extraction solution. In addition, the electrical conductivity of beet tissue after application of 20 pulses at 4.38 kV/cm was significantly higher compared to the untreated sample (Figure 1). 

In the case of beetroot pulp, an increase in betanin content (Figure 2), even if not statistically significant, can be observed, as well as a decrease in vulgaxanthin extractability (statistically significant) with increasing intensity of the electric field (Figure 3). In addition, the fragmentation of the raw material tissue significantly increased the content of both betanin and vulgaxanthin in the extract, compared to their content in the beetroot cylinders. The highest content of vulgaxanthin was observed in the extract of untreated pulp (control), while the lowest was after the treatment with the electric field of 6.25 kV/cm and 30 pulses, which caused statistically significant (*p* < 0.05) drop in vulgaxanthin content by 27%. In turn, in the case of betanin, an inverse relationship was demonstrated, in the PEF application with the parameters E = 6.25 kV/cm, *n* = 30 promoted the higher extraction efficiency, increased by 16% in relation to the reference sample; however, this was not statistically significant. 

Information on the influence of PEF on the vulgaxanthin content in the literature is really scarce. Nevertheless, the decrease in its content in the beetroot pulp, along with the increasing intensity of the electric field, could be related to the physicochemical properties of this pigment. Vulgaxanthin is less stable in an acid environment and at room temperature (and higher) compared to betanin. The solvent used in the test was a slightly acidic buffer (pH = 6.5), and in addition, although PEF treatment is a non-thermal method, the temperature was slightly risen during its application. In addition, the fragmentation of the material facilitated the extraction of pigment from the tissue, which resulted in longer exposure of vulgaxanthin to a temperature higher than room temperature, thus it could cause its degradation [29].

The use of electrical treatment allows achievement of a high degree of disintegration of cells with only small temperature increases (up to 9.1 °C). Two examples in the literature deal with the identification of the optimal amount of energy to maximize pigment extraction from beetroot tissue (between 2.5 and 7 kJ/kg obtained using a different electric field intensity). For example, a PEF treatment at 1 kV/cm (270 pulses, 10 μs, 7 kJ/kg) allowed to release about 90% of the total red pigment after 1 h of extraction compared to less than 5% in the untreated samples [17,30]. López et al. [18] instead observed that the application of 7 kV/cm (5 pulses of 2 μs, 2.5 kJ/kg) caused a release of about 90% of total betanin from beetroot samples in 300 min. This release was 5-fold quicker than the control samples, while the application of higher electric field strength (9 kV/cm) decreased the extraction yield of the betanin. In this case, the energy supplied to the beetroot was lower than in the present study and in the work of Fincan et al. [17]. 

### 3.4. HCA and Pearson’s Correlation

**Figure 4:**
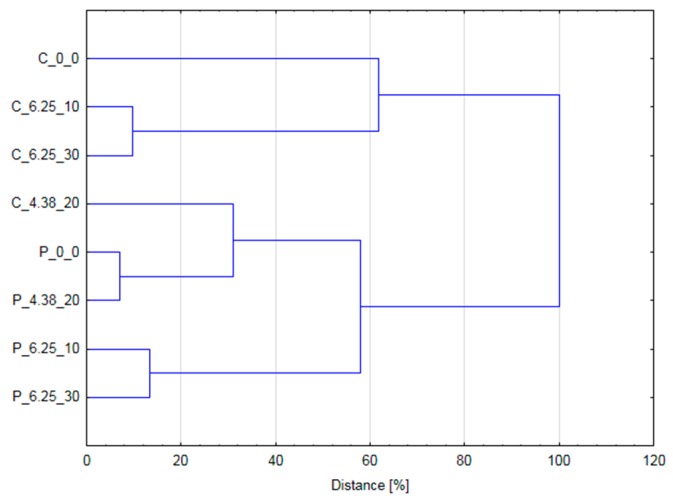
The results of hierarchical cluster analysis (HCA) performed using color parameters (L*, a*, b*) and concentration of betanin and vulgaxanthin. (C_0_0 = untreated beetroot cylinders; C_4.38_20 = beetroot cylinders treated by 20 pulses at 4.38 kV/cm; C_6.25_10 = beetroot cylinders treated by 10 pulses at 6.25 kV/cm; C_6.25_30 = beetroot cylinders treated by 30 pulses at 6.25 kV/cm; P_0_0 = untreated beetroot pulp; P_4.38_20 = beetroot pulp treated by 20 pulses at 4.38 kV/cm; P_6.25_10 = beetroot pulp treated by 10 pulses at 6.25 kV/cm; P_6.25_30 = beetroot pulp treated by 30 pulses at 6.25 kV/cm).

Figure 4 presents the results of HCA, which was carried out using normalized color data (L*, a* and b*) and concentration of betanin and vulgaxanthin. Performed analysis allowed the samples to be grouped in two big clusters. The first group consisted of most of the samples in cylindrical form (C_0_0; C_6.25_10; C_6.25_30), whereas the second group included all samples in pulp form and one cylindrical sample—the one treated with 20 pulses at 4.38 kV/cm. This sample formed a smaller aggregation that included untreated pulp form beetroot (P_0_0) and the pulp form material treated by the same parameters. Hence, it can be stated that PEF treatment can lead to similar changes in the tissue brought about by PEF influencing extraction are similar to the changes due to the mechanical disintegration obtained by pulping. Beetroot samples treated with 6.25 kV/cm were grouped in two separate aggregates depending on their physical form, cylinder or pulp. This information is very valuable because it indicates that increasing the number of pulses does not bring additional benefits. Such a behavior in literature is defined as ‘saturation level of electroporation’, meaning that no more changes can be achieved by PEF treatment despite of utilization of higher energy input [31].

The results of Pearson’s correlation analysis of selected variables are presented in Table 4. Significant correlation has been found between lightness (L*) and a* coordinate. What is more interesting is that both L* and a* values presented a significant relationship with betanin and vulgaxanthin concentration. However, higher values of Pearson’s correlation coefficient were found between color parameters and betanin concentration. Similar observations were also reported by Antigo et al. [32], who examined the betanin content of microcapsules of beetroot extract obtained by spray drying and freeze-drying. 

## 4. Conclusions

The use of PEF led to an increase of the electrical conductivity of beetroot tissue, thus leading to an increase of the cell membrane permeability. When lower electric field intensity (4.38 kV/cm) was applied, the electrical conductivity increased proportionally to the pulse number applied, whereas with the higher intensity (6.25 kV/cm) this effect was negligible, showing an effect of saturation level of electroporation. PEF treatment caused changes in the color of red beetroot tissue that were associated with a better extraction of pigments as a result of the electroporation. The use of PEF with an intensity of 4.38 kV/cm and 20 impulses and energy consumption of 4.10 kJ/kg, allowed an increase in the yield of betanin and vulgaxanthin extraction from beetroot cylinders by 329% and 244%, respectively, compared to the control. Furthermore, the fragmentation of the tissue (pulping) resulted in an increased extraction of pigments from the plant tissue; however, the use of PEF did not bring additional advantages.

## Figures and Tables

**Table 1:** Parameters of pulsed electric field (PEF) treatment: electric field intensity, pulse number, and energy delivered to the sample used in the investigation.

Sample Code	Electric Field Intensity (kV/cm)	Pulse Number (-)	Energy (kJ/kg)
0_0	0	0	0
4.38_10	4.38	10	2.43
4.38_20	4.38	20	4.86
4.38_30	4.38	30	7.28
6.25_10	6.25	10	4.96
6.25_20	6.25	20	9.92
6.25_30	6.25	30	14.88

**Table 2:** Color parameters (L* = lightness, a* = red/green index, b* = yellow/blue index) of untreated and PEF-treated beetroot cylinders before and after extraction.

Sample	L*		a*		b*	
	Before	After	Before	After	Before	After
0_0	21.1 ± 0.8 ^a^	21 ± 1 ^AB^	27 ± 2 ^a^	29 ± 2 ^A^	7.74 ± 0.6 ^a^	8.8 ± 0.9 ^A^
4.38_20	18.9 ± 0.9 ^c^	20.7 ± 0.7 ^A^	18 ± 3 ^c^	20 ± 2 ^B^	4.6 ± 0.9 ^c^	4.9 ± 0.7 ^C^
6.25_10	20 ± 1 ^b^	24.5 ± 0.8 ^C^	23 ± 3 ^b^	31 ± 2 ^A^	6 ± 1 ^b^	6.1 ± 0.9 ^BC^
6.25_30	18 ± 1 ^c^	23 ± 1 ^BC^	18 ± 2 ^c^	29 ± 3 ^A^	4.6 ± 0.8 ^c^	7 ± 1 ^B^

The same letter on the same column means no significant difference by the Tukey test (*p* < 0.05).

**Table 3:** Color parameters (L* = Lightness, a* = red/green index, b* =yellow/blue index) of extracts obtained from untreated and PEF-treated beetroot cylinders and pulp.

Sample	L*		a*		b*	
	Cylinders	Pulp	Cylinders	Pulp	Cylinders	Pulp
0_0	96.2 ± 0.2 ^a^	72 ± 1 ^A^	7.2 ± 0.4 ^a^	52 ± 2 ^A^	−0.6 ± 0.1 ^a^	−1.7 ± 0.4 ^A^
4.38_20	76 ± 3 ^b^	70 ± 2 ^A^	43 ± 5 ^b^	55 ± 4 ^A^	1.5 ± 1.6 ^a^	−1.4 ± 0.5 ^A^
6.25_10	81 ± 2 ^b^	70 ± 1 ^A^	36 ± 4 ^b^	57 ± 2 ^A^	−5 ± 2 ^b^	−6.16 ± 0.06 ^B^
6.25_30	79 ± 2 ^b^	70 ± 2 ^A^	41 ± 3 ^b^	58 ± 4 ^A^	−5.1 ± 0.7 ^b^	−9.3 ± 06 ^C^

The same letter on the same column means no significant difference by the Tukey test (*p* < 0.05).

**Table 4:** The results of Pearson’s correlation analysis.

Variable	L*	a*	b*	BC	VC
L*	-	*r* = −0.9977	*r* = 0.3593	*r* = −0.9760	*r* = −0.8853
		*p* < 0.001	*p* = 0.382	*p* < 0.001	*p* = 0.003
a*	*r* = −0.9977	-	*r* = −0.4214	*r* = 0.9821	*r* = 0.8594
	*p* < 0.001		*p* = 0.298	*p* < 0.001	*p* = 0.006
b*	*r* = 0.3593	*r* = −0.4214	-	*r* = −0.4692	*r* = 0.0077
	*p* = 0.382	*p* = 0.298		*p* = 0.241	*p* = 0.986
**BC**	*r* = −0.9760	*r* = 0.9821	*r* = −0.4692	-	*r* = 0.8623
	*p* < 0.001	*p*<0.001	*p* = 0.241		*p* = 0.006
**VC**	*r* = −0.8853	*r* = 0.8594	*r* = 0.0077	*r* = 0.8623	-
	*p* = 0.003	*p* = 0.006	*p* = 0.986	*p* = 0.006	

*p*-Values lower than 0.05 indicate the significant character of the correlation between normalized variables. L*, a*, b* = color parameters of the extracts; BC = betanin concentration in the sample; VC = vulgaxanthin concentration in the sample.

## References

[1] Wruss J., Waldenberger G., Huemer S., Uygun P., Lanzerstorfer P., Müller U., Höglinger O., Weghuber J. (2015). Compositional characteristics of commercial beetroot products and beetroot juice prepared from seven beetroot varieties grown in Upper Austria. J. Food Compos. Anal..

[2] Chhikara N., Kushwaha K., Sharma P., Gat Y., Panghal A. (2019). Bioactive compounds of beetroot and utilization in food processing industry: A critical review. Food Chem..

[3] Slavov A., Karagyozov V., Denev P., Kratchanova M., Kratchanov C. (2013). Antioxidant activity of red beet juices obtained after microwave and thermal pretreatments. Czech J. Food Sci..

[4] Strack D., Vogt T., Schliemann W. (2003). Recent advances in betalain research. Phytochemistry.

[5] Gengatharan A., Dykes G.A., Choo W.S. (2015). Betalains: Natural plant pigments with potential application in functional foods. LWT—Food Sci. Technol..

[6] Szopinska A.A., Gawęda M. (2013). Comparison of yield and quality of red beet roots cultivated using conventional, integrated and organic method. J. Hort. Res..

[7] Tanabtabzadeh M.S., Javanbakht V., Golshirazi A.H. (2019). Extraction of Betacyanin and Betaxanthin Pigments from Red Beetroots by Chitosan Extracted from Shrimp Wastes. Waste Biomass Valoriz..

[8] Celli G.B., Brooks M.S.-L. (2017). Impact of extraction and processing conditions on betalains and comparison of properties with anthocyanins—A current review. Food Res. Int..

[9] Singh A., Ganesapillai M., Gnanasundaram N. (2017). Optimizaton of extraction of betalain pigments from *beta vulgaris* peels by microwave pretreatment. IOP Conference Series: Materials Science and Engineering.

[10] Cardoso-Ugarte G.A., Sosa-Morales M.E., Ballard T., Liceaga A., San Martín-González M.F. (2014). Microwave-assisted extraction of betalains from red beet (*Beta vulgaris*). LWT—Food Sci. Technol..

[11] Ramli N.S., Ismail P., Rahmat A. (2014). Influence of conventional and ultrasonic-assisted extraction on phenolic contents, betacyanin contents, and antioxidant capacity of red dragon fruit (Hylocereus polyrhizus). The Sci. World J..

[12] Laqui-Vilca C., Aguilar-Tuesta S., Mamani-Navarro W., Montaño-Bustamante J., Condezo-Hoyos L. (2018). Ultrasound-assisted optimal extraction and thermal stability of betalains from colored quinoa (Chenopodium quinoa Willd) hulls. Ind. Crop. Prod..

[13] Tylewicz U., Tappi S., Mannozzi C., Romani S., Dellarosa N., Laghi L., Ragni L., Rocculi P., Dalla Rosa M. (2017). Effect of pulsed electric field (PEF) pre-treatment coupled with osmotic dehydration on physico-chemical characteristics of organic strawberries. J. Food Eng..

[14] Wiktor A., Sledz M., Nowacka M., Rybak K., Chudoba T., Lojkowski W., Witrowa-Rajchert D. (2015). The impact of pulsed electric field treatment on selected bioactive compound content and color of plant tissue. Innov. Food Sci. Emerg. Technol..

[15] Vorobiev E., Lebovka N.I. (2010). Enhanced extraction from solid foods and biosuspensions by pulsed electrical energy. Food Eng. Rev..

[16] Barba F.J., Parniakov O., Pereira S.A., Wiktor A., Grimi N., Boussetta N., Saraiva J.A., Raso J., Martin-Belloso O., Witrowa-Rajchert D. (2015). Current applications and new opportunities for the use of pulsed electric fields in food science and industry. Food Res. Int..

[17] Fincan M., DeVito F., Dejmek P. (2004). Pulsed electric field treatment for solid-liquid extraction of red beetroot pigment. J. Food Eng..

[18] López N., Puértolas E., Condón S., Raso J., Alvarez I. (2009). Enhancement of the extraction of betanine from red beetroot by pulsed electric fields. J. Food Eng..

[19] Luengo E., Martinez J.M., Álvarez I., Raso J. Comparison of the efficacy of pulsed electric fields treatments in the millisecond and microsecond range for the extraction of betanine from red beetroot. Proceedings of the 1st World Congress on Electroporation and Pulsed Electric Fields in Biology, Medicine and Food & Environmental Technologies (WC 2015).

[20] Chalermchat Y., Fincan M., Dejmek P. (2004). Pulsed electric field treatment for solid-liquid extraction of red beetroot pigment: mathematical modelling of mass transfer. J. Food Eng..

[21] Wiktor A., Iwaniuk M., Śledź M., Nowacka M., Chudoba T., Witrowa-Rajchert D. (2013). Drying kinetics of apple tissue treated by pulsed electric field. Dry Technol..

[22] Fijalkowska A., Nowacka M., Witrowa-Rajchert D. (2017). The physical, optical and reconstitution properties of apples subjectes to ultrasound before drying. Ital. J. Food Sci..

[23] Fijalkowska A., Nowacka M., Witrowa-Rajchert D. (2015). The influence of ultrasound pre-treatment on drying kinetics and the colour and betalains content in beetroot. Zeszyty Problemowe Postępów Nauk Rolniczych.

[24] Lebovka N., Bazhal M., Vorobiev E. (2002). Estimation of characteristic damage time of food materials in pulsed electric fields. J. Food Eng..

[25] Tylewicz U., Aganovic K., Vannini M., Toepfl S., Bortolotti V., Dalla Rosa M., Oey I., Heinz V. (2016). Effect of pulsed electric field treatment on water distribution of freeze-dried apple tissue evaluated with DSC and TD-NMR techniques. Innov. Food Sci. Emerg. Technol..

[26] Lebovka N.I., Bazhal M.I., Vorobiev E. (2000). Simulation and experimental investigation of food material breakage using pulsed electric field treatment. J. Food Eng..

[27] Bazhal M., Lebovka N., Vorobiev E. (2003). Optimisation of pulsed electric field strength for electroplasmolysis of vegetable tissues. Biosyst. Eng..

[28] Puértolas E., Saldaña G., Raso J., Miklavčič D. (2017). Pulsed electric field treatment for fruit and vegetable processing. Handbook of Electroporation.

[29] Manchali S., Murthy K.N.C., Nagaraju S., Neelwarne B., Neelwarne B. (2013). Stability of Betalain Pigments of Red Beet. Red Beet Biotechnology: Food and Pharmaceutical Applications.

[30] Donsì F., Ferrari G., Pataro G. (2010). Applications of pulsed electric field treatments for the enhancement of mass transfer from vegetable tissue. Food Eng. Rev..

[31] Canatella P.J., Karr J.F., Petros J.A., Prausnitz M.R. (2001). Quantitative study of electroporation-mediated molecular uptake and cell viability. Biophys. J..

[32] Antigo J.L.D., Bergamasco R.D.C., Madrona G.S. (2018). Effect of pH on the stability of red beet extract (*Beta vulgaris* L.) microcapsules produced by spray drying or freeze drying. Food Sci. Technol..

